# Prolonged ECMO support in a pediatric patient with complex cardiac conditions during wartime in Kyiv, Ukraine

**DOI:** 10.1051/ject/2025004

**Published:** 2025-03-07

**Authors:** Daniel Rusnak, Ignazio Condello, Valerii Dohtiar, Serhii Varbanets, Kristina Alieksieieva, Serhii Luhovkin, Vladyslav Palokha, Vadym Zhoha, Neonila Bukhnei, Lesia Krashevska, Olha Kutsenko, Nataliia Kupina, Oleksii Vnukov

**Affiliations:** 1 The Scientific and Practical Medical Center of Pediatric Cardiology and Cardiac Surgery of the Ministry of Health of Ukraine Київ, вул. В'ячеслава Чорновола 28/1; 2 Department of Cardiac Surgery, Anthea Hospital, GVM Care & Research Via Camillo Rosalba 35/37 70124 Bari Italy

**Keywords:** Extracorporeal membrane oxygenation (ECMO), Pediatric critical care, Prolonged ECMO support, Congenital heart disease, Aortic stenosis, Myocarditis, Conflict zone healthcare, Polypropylene Oxygenators, Oxygenator failure, Resource-limited settings, Organ transplantation barriers, War-affected medical care, ECMO complications, Supply chain limitations, Cardiopulmonary support

## Abstract

*Background*: In the conflict-affected setting of Kyiv, Ukraine, managing complex medical interventions presents significant challenges, especially for critically ill pediatric patients. This case report describes the prolonged use of extracorporeal membrane oxygenation (ECMO) in a 12-year-old girl with severe congenital heart disease, including critical aortic stenosis and myocarditis. In the absence of a transplant system and with limited resources, alternative solutions were explored to balance the high risk of complications and maintain ECMO support over an extended period. *Methods*: The patient received ECMO support for a total of 259 days, utilizing both VV-ECMO and VA-ECMO configurations. Due to wartime supply issues, polypropylene fiber oxygenators, specifically the Quadrox-i and Terumo Fx15 models, were used despite not being indicated for long-term use. Eleven oxygenators were employed, with a total of ten replacements due to thrombosis and technical malfunctions. Oxygenator models included EUROSETS Alone, Maquet PLS, Paragon, Terumo Fx15, and Quadrox-i. Frequent adjustments to ECMO parameters, infection control, and daily rehabilitation efforts were essential components of care. Weaning tests consistently indicated no recovery of cardiac function. *Results*: Despite rigorous management, the oxygenators faced performance declines over time, including clotting, reduced oxygen exchange, and lower CO_2_ removal efficiency, necessitating frequent replacements, with a total of ten changes across the ECMO course. Oxygenator usage durations were as follows: EUROSETS (10, 32, 88, and 26 days), Paragon (78 days), Maquet PLS (14 days), Terumo Fx15 (5, 1, 3, and 2 days), and Quadrox-i (1 day). Notably, EUROSETS nr 3 achieved the longest duration of 88 days, while the Paragon oxygenator provided stable support for 78 days. In contrast, the Maquet PLS oxygenator lasted 14 days, and the Terumo Fx15 and Quadrox-i models required rapid replacement within 1–5 days due to accelerated wear and reduced efficacy. Ultimately, ECMO support was discontinued due to irreversible heart failure, with limited options for heart-lung transplantation in Ukraine’s conflict-impacted healthcare system. The patient passed away following the cessation of ECMO support due to severe multi-organ dysfunction and complications. *Conclusions*: This case underscores the extraordinary challenges of prolonged ECMO use in a pediatric patient within a war zone, highlighting the resilience and adaptability required of healthcare teams in resource-limited settings. The logistical constraints imposed by a lack of suitable biomedical devices and the necessity to use suboptimal models due to supply shortages emphasize the need for international support and resource mobilization to sustain advanced medical care in conflict-affected regions.

## Background

The ongoing war in Ukraine has caused severe and widespread disruption to healthcare systems, particularly in heavily affected areas such as Kyiv. Healthcare facilities, already strained by the high demand for trauma care, face drastic shortages of essential medical supplies, equipment, and skilled personnel [1]. In this conflict-driven context, hospitals are often forced to function with limited resources, as supply chains for critical medical equipment are severely hampered by barriers such as damaged infrastructure, restricted transportation, and interrupted international support. These limitations drastically affect the provision of complex and resource-intensive care, especially for vulnerable populations like pediatric patients who require specialized interventions. The need for durable, reliable medical devices is especially critical under such conditions, where resource scarcity makes frequent replacements or repairs nearly impossible [1]. This necessity is particularly pronounced for life-sustaining interventions such as extracorporeal membrane oxygenation (ECMO), which requires robust oxygenator performance to ensure patient survival over extended periods. In a stable setting, ECMO systems rely on high-quality, long-lasting devices to maintain consistent gas exchange and minimize complications [2]. However, in Kyiv’s war-torn environment, access to the most advanced and durable devices is significantly restricted. Limited availability has led to the use of suboptimal devices, such as polypropylene fiber oxygenators (Quadrox-i and Terumo Fx15 models), which, while effective in the short-term, are not recommended for long-term use due to their tendency for rapid wear and reduced efficiency in oxygen exchange and CO_2_ removal. These forced compromises in device selection underscore the critical need for efficient and durable biomedical equipment in conflict settings, where routine maintenance and device replacement can be prohibitively challenging. In the absence of reliable supply chains, healthcare providers are often unable to replace malfunctioning devices promptly, which can lead to increased patient risk [1]. The frequent device failures associated with using less durable equipment strain limited resources, as each oxygenator replacement requires additional time, technical expertise, and materials that are in short supply. This report details the case of a 12-year-old girl with severe congenital heart disease and myocarditis, who underwent prolonged ECMO support for 259 days in Kyiv amidst these constraints. Her case highlights the extraordinary challenges faced by healthcare providers in sustaining ECMO support in a setting with significant supply limitations, where barriers to obtaining efficient and durable medical equipment greatly impacted patient care and outcomes. The experience reflects the broader need for international collaboration and support in supplying conflict-affected healthcare systems with the necessary resources to deliver complex, life-sustaining care amidst persistent logistical and resource challenges.

## Materials and methods

### Patient information

The patient was a 12-year-old girl, height 144 cm, weight 25 kg, and body surface area (BSA) 1.4 m^2^, with severe congenital heart disease. Her diagnosis included critical aortic valve stenosis with a mono-cuspid aortic valve, moderate mitral valve insufficiency, and a partial anomalous pulmonary venous connection (right superior pulmonary vein connected to the superior vena cava). She presented with severe heart failure, indicated by an ejection fraction of 17%, acute myocarditis, acute respiratory distress syndrome (ARDS), pneumonia, bilateral hydrothorax, and ascites. Upon transfer to our clinic on January 28, 2024, her critical status necessitated immediate interventions. Two endovascular balloon valvuloplasties were performed early on January 29, with minimal improvement. By evening, with a Murray score of 3.25, VV-ECMO was initiated to stabilize her respiratory and cardiac function. Shortly after, she tested positive for Influenza A, further complicating her condition. Over the next few days, her condition remained critical. Additional treatments, including continuous renal replacement therapy (CRRT) and inotropic support optimization, were implemented. Given her declining hemodynamics, she underwent aortic valve replacement with a mechanical prosthesis, followed by a shift to VA-ECMO due to worsening cardiac function. Myocardial biopsy confirmed myocarditis, leading to her placement on the heart-lung transplant waiting list. Unfortunately, the absence of compatible donors due to logistical constraints prevented any further transplantation interventions. This study was conducted in accordance with the ethical standards of the institutional and national research committee. Approval for this study and its publication was obtained from the Internal Institutional Review Board (IRB), ensuring all ethical concerns regarding patient treatment and data confidentiality were thoroughly addressed. The manuscript has been checked for conflicts of interest, and none were found that could affect the integrity of the research.

### Operative plan

**Figure 1:**
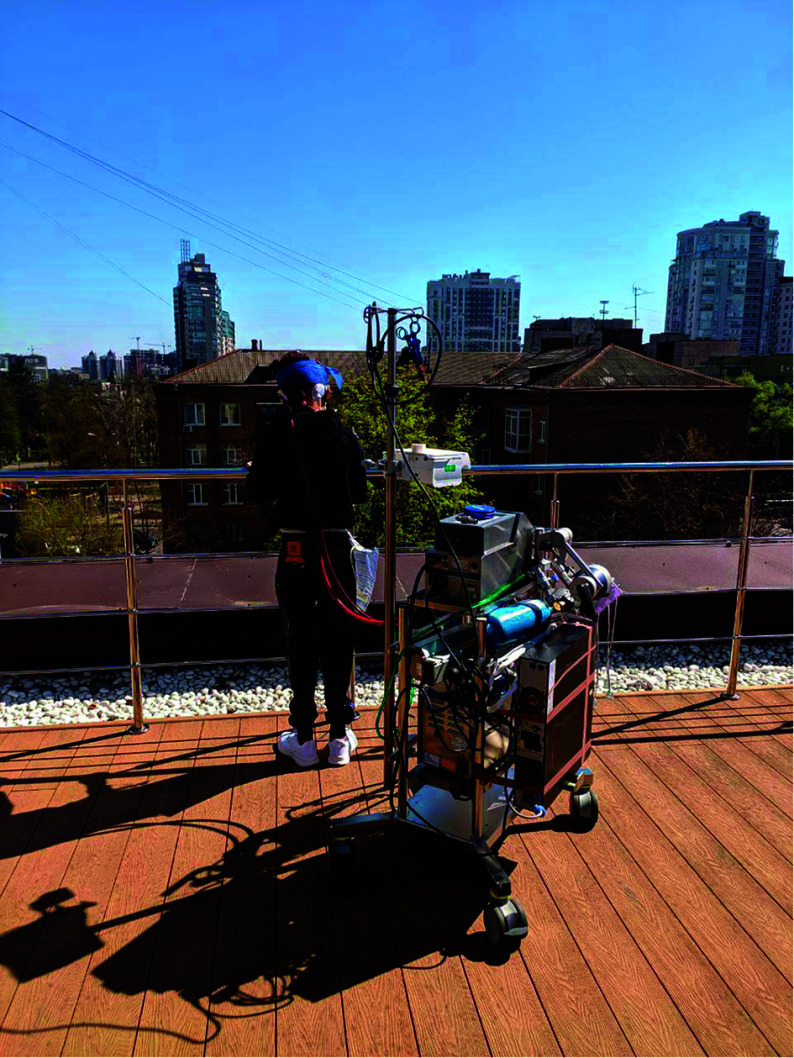
Patient Rehabilitation during VA ECMO.

The ECMO course for this 12-year-old patient spanned a prolonged and complex 259 days, necessitated by severe respiratory and cardiac complications and marked by frequent adjustments to both the ECMO configuration and oxygenator components. Initially, VV-ECMO (Femoral vein Maquet 23Fr and Jugular vein HLS 19Fr), was initiated on January 29, 2024, due to the patient’s respiratory distress and hemodynamic instability. Given the persistence of cardiac compromise and eventual deterioration in hemodynamics, the ECMO was transitioned to VA-ECMO on February 8, 2024. Cannulation for VA-ECMO involved the use of a Maquet 23F cannula for drainage in the right internal jugular vein and an ALS 17F cannula for arterial return in the common carotid artery, utilizing a vascular prosthesis. These adjustments allowed for greater support to both the heart and lungs throughout the ECMO course, with additional modifications made as required by the patient’s evolving condition. The patient’s clinical history included congenital heart disease with severe aortic valve stenosis, moderate mitral valve insufficiency, partial anomalous pulmonary venous connection, and advanced heart failure (EF LV-17%). She had been hospitalized on January 28, 2024, with a suspected diagnosis of acute myocarditis, alongside ARDS, bilateral pneumonia, and complications such as hydrothorax and ascites. Influenza A was also confirmed shortly after admission. Initial interventions included two endovascular balloon valvuloplasties on January 29, 2024, performed to relieve the severe aortic stenosis prior to ECMO initiation. Following ECMO initiation, a CRRT was introduced to manage fluid balance and optimize inotrope therapy. The patient underwent an aortic valve replacement (AVR) on February 2, 2024, during which the ECMO circuit was converted to cardiopulmonary bypass (CPB), and a myocardium biopsy confirmed myocarditis. As part of long-term respiratory support, a tracheostomy was performed on February 13, 2024. The patient was also managed on ECMO-awake protocols, engaging in daily mobilization and rehabilitation, which included ambulation within the hospital, despite being on ECMO support (Figure 1). Supply shortages during the conflict in Kyiv necessitated the use of polypropylene-based oxygenators (such as Quadrox-i and Terumo Fx15), which are typically unsuitable for long-term ECMO due to their propensity for premature failure. This led to an increased frequency of oxygenator replacements, totaling ten changes over the course of treatment. The EUROSETS oxygenators demonstrated relatively prolonged usage durations, and low levels of plasma free hemoglobin (Table 1), including the longest span of 88 days with Eurosets oxygenator nr 3, while Terumo Fx15 oxygenators had to be replaced within a maximum of 2 days due to high transmembrane pressures. Later in the course, PLS Quadrox and Paragon PMP oxygenators were used, with Paragon sustaining usage for 79 days. Regular adjustments to the ECMO circuit were also essential, including a complete circuit and cannula replacement on June 7, 2024, due to extensive thrombosis and mechanical complications. On July 3, 2024, the patient experienced acute thrombosis of the oxygenator, requiring immediate replacement with a Terumo Fx15 unit, and further replacements were subsequently made. The ECMO was operated in VA-ECMO mode with flows between 2000 and 2300 mL/min. Typical pressure readings included pre-oxygenator pressures of 220 mmHg, post-oxygenator pressures of 185 mmHg, and venous pressures around 30 mmHg. Maximum lactate levels reached 6 mmol/L, reflecting the patient’s metabolic status throughout the ECMO support. Daily weaning tests were conducted, although all results remained negative. The pulmonary function recovered remarkably well despite multiple episodes of bleeding, confirmed by favorable pulmonary vein measurements during a subsequent angio-operation. However, the heart remained compromised with severely reduced function, reflected by a persistently low left ventricular outflow tract velocity time integral (LVOT VTI) and Ejection Fraction (EF), underscoring the need for a heart-lung transplant. Unfortunately, due to the limited transplant infrastructure in the country, compounded by the war’s impact, no suitable donor became available. On October 14, 2024, ECMO support was discontinued following system dysfunction and deteriorating patient stability. Despite the extensive, multidisciplinary effort to sustain this young patient, she ultimately succumbed to multiorgan dysfunction secondary to her underlying cardiac failure.

**Table 1:** Performance and duration of each oxygenator models used during the ECMO course, are presented as mean values and standard deviation.

Parameter	EUROSETS nr1	EUROSETS nr2	EUROSETS nr3	EUROSETS nr4	Maquet PLS	Paragon PMP	Terumo Fx15 nr1	Terumo Fx15 nr2	Terumo Fx15 nr3	Terumo Fx15 nr4	Quadrox-i
Days in use	10	32	88	26	14	78	5	1	3	2	1
Hb Mean (SD)	11.43 ± 0.98	10.34 ± 0.73	10.75 ± 0.87	11.53 ± 0.71	11.75 ± 0.54	11.17 ± 1.01	9.96 ± 1.11	9.4 ± ~1	11.67 ± 1.07	12.85 ± 1.2	12.7
PaO_2_ post ox Mean (SD) [mmHg]	419 ± ~50	289.55 ± 55.66	310.67 ± 52.32	320 ± 43.84	128 ± 96.17	325.78 ± 75.58	287 ± ~50	346 ± ~50	243 ± ~50	399 ± ~50	~300 ± ~50
PaCO_2_ postox Mean (SD) [mmHg]	39.9 ± ~5	32.8 ± 5.71	33.03 ± 1.92	35 ± 1.41	29 ± 1.41	30.36 ± 5.92	30 ± ~5	30.3 ± ~5	40 ± ~5	34 ± ~5	~35 ± ~5
Air (L/min) Mean (SD)	2.25 ± 0.26	1.64 ± 1.01	2.03 ± 0.92	1.99 ± 1.38	2.71 ± 1.79	1.71 ± 0.40	2.8 ± 1.1	2 ± ~0.5	2.4 ± ~0.5	3 ± ~0.5	4 ± ~0.5
FiO_2_ (%) Mean (SD)	100 ± 0	71.09 ± 18.13	70 ± 20.68	71.54 ± 23.78	79.29 ± 18.99	70.26 ± 15.87	100 ± 0	100 ± 0	100 ± 0	100 ± 0	100 ± 0
V′O_2_ Mean (SD)	131 ± N/A	104 ± 11.92	104.13 ± 2.64	115 ± 1.41	103.5 ± 13.44	110.33 ± 12.53	N/A	N/A	N/A	N/A	N/A
DO_2_ Mean (SD)	572 ± N/A	333 ± 39.50	331.67 ± 14.80	396.5 ± 10.61	353 ± 26.87	349.33 ± 31.88	N/A	N/A	N/A	N/A	N/A
BFR (L/min) Mean (SD)	3.31 ± 0.09	2.10 ± 0.11	2.15 ± 0.13	2.06 ± 0.21	2.20 ± N/A	2.20 ± N/A	2.31 ± 0.03	2.30 ± N/A	2.40 ± 0.05	2.23 ± 0.04	2.70 ± N/A
Pump RPM Mean (SD)	3050 ± 65.4	3120 ± 46.84	3101.14 ± 71.49	3100 ± 45.34	3130 ± 33.40	3100 ± 88.20	3250 ± 45.18	3250 ± 33.56	3250 ± 28.23	3050 ± 35.45	3150 ± 45.23
Pressure drop Mean (SD) [ΔP]	62.9 ± 1.97	53.09 ± 7.86	56.69 ± 8.07	65.54 ± 27.04	47.36 ± 18.83	45.32 ± 5.34	32 ± 10	35 ± 10	34 ± 10	41 ± 10	35 ± 10
Arterial blood temperature Mean (°C)	37.0	37.0	37.0	37.0	37.0	37.0	37.0	37.0	37.0	37.0	37.0
Venous blood temperature Mean (°C)	36.8	36.8	36.8	36.8	36.8	36.8	36.8	36.8	36.8	36.8	36.8
Continuous anticoagulation use	Yes	Yes	Yes	Yes	Yes	Yes	Yes	No	No	No	No
DDs (μg/mL)	150 ± 30	~00 ± 40	~90 ± 35	210 ± 50	180 ± 45	170 ± 38	160 ± 25	300 ± 60	220 ± 40	250 ± 55	320 ± 65
LDH Mean (SD) [IU/L]	1097.57 ± 328.67	535.83 ± 37.75	572.88 ± 39.01	700 ± ~100	2181.5 ± 1892.78	339.29 ± 48.26	~500 ± 67	5000 ± 89	500 ± 74	2224 ± 123	5043 ± 114
Fibrinogen Mean (mg/dL)	155 ± 7.07	355.5 ± 128.75	286.25 ± 74.31	N/A	N/A	138.88 ± 37.51	N/A	N/A	N/A	N/A	N/A
PLT Mean (10⁹/L)	108.7 ± 27.61	144.44 ± 37.36	173.36 ± 43.75	156.65 ± 42.79	125.86 ± 27.15	101.72 ± 35.16	124.6 ± 18.85	129 ± N/A	N/A	85 ± 7.07	120 ± N/A
aPTT Mean (s)	58.5 ± 24.74	53.91 ± 12.74	64.23 ± 25.95	64.92 ± 25.34	54.36 ± 18.76	N/A	N/A	N/A	N/A	31 ± N/A	N/A
Free Hb Mean (SD) [mg/dL]	124.44 ± 33.58	77.19 ± 16.11	61.34 ± 13.01	80 ± 20	100 ± 30	100 ± 25	80 ± 20	80 ± 20	80 ± 20	180 ± 20	510 ± 50

### Management of anticoagulation and blood products

At our center, patients undergoing ECMO therapy are managed with a continuous infusion of heparin. The target for activated partial thromboplastin time (aPTT) is set between 50 s and 65 s, unless specific clinical conditions, such as active bleeding, necessitate alternative parameters. The heparin dose is adjusted using a nurse-driven protocol based on a nomogram. This approach considers the patient’s weight to determine the initial infusion rate. An aPTT measurement is obtained six hours after starting the infusion. Based on the result, the infusion rate is modified: increased if the aPTT is below 50 s, decreased if above 65 s, or maintained if within the target range. Subsequent aPTT checks are performed every 6 h until two consecutive results are within the target range, after which daily monitoring is instituted. For platelet management, the standard practice at our institution involves transfusion when platelet counts drop below 80,000/μL. However, some advanced centers adopt a more conservative threshold, intervening only when levels fall to 40,000–50,000/μL or as low as 20,000/μL in patients who are not actively bleeding. Red blood cell transfusion strategies vary based on hemoglobin (Hb) levels and physiological factors such as blood flow (BF), cardiac output (CO), and oxygen delivery (DO_2_). Restrictive transfusion protocols are applied when Hb levels fall within 7–9 g/dL, whereas a more liberal approach is taken for Hb levels of 10–12 g/dL [9]. In this case report, the restrictive blood transfusion protocol was implemented driven by the need to conserve scarce resources highlights the dire supply situation that dictates every aspect of ECMO management in a conflict-affected setting.

## Results

**Figure 2:**
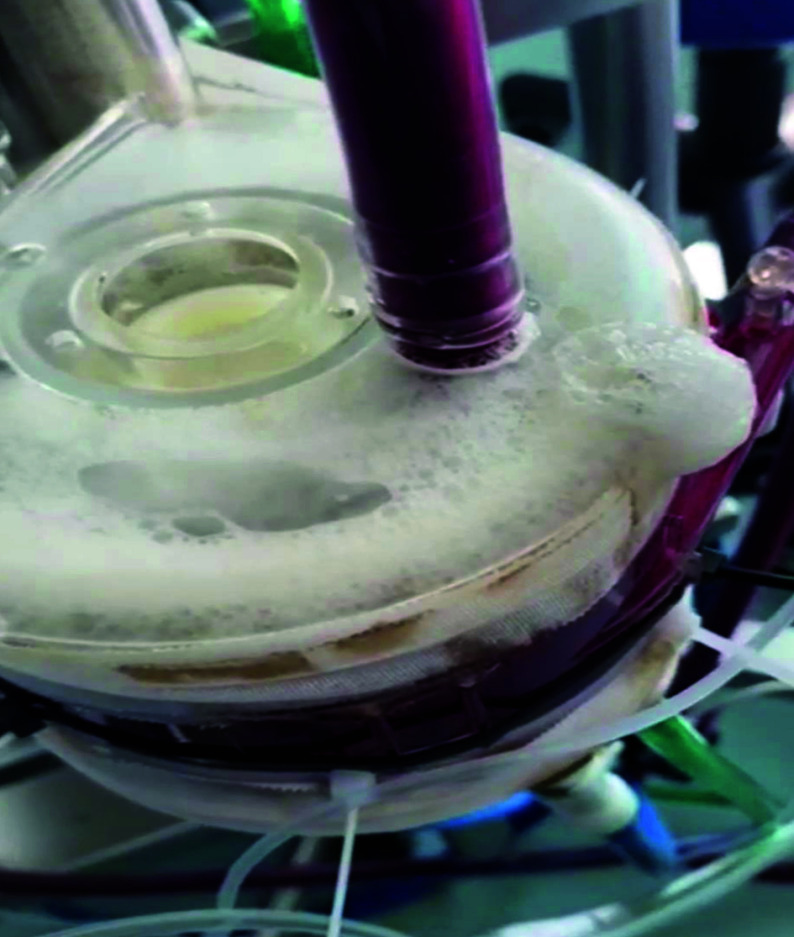
Plasma Leakage on Polypropylene Oxygenator.

**Figure 3:**
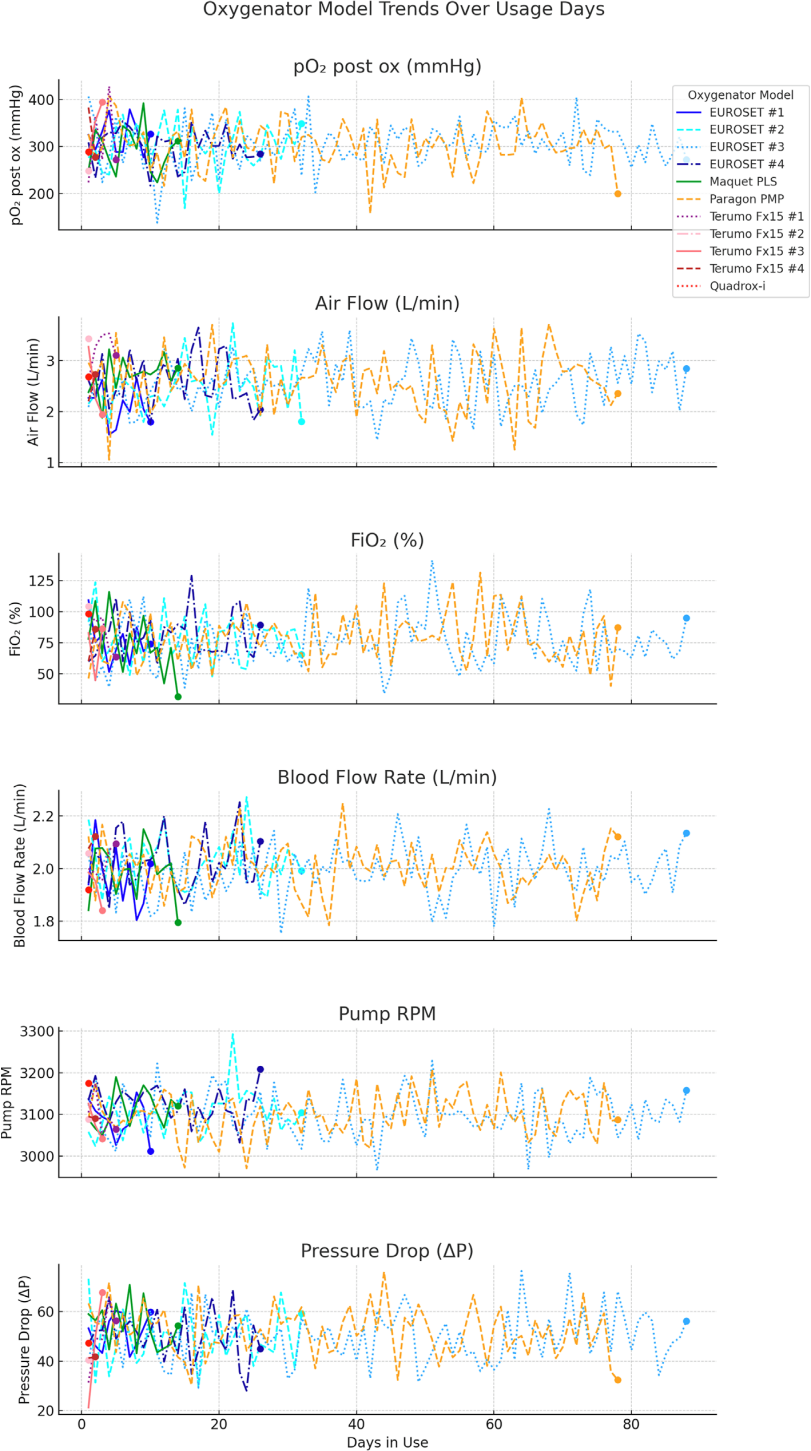
Oxygenator models and values trends.

Throughout the 259-day ECMO course, a total of 11 oxygenators were utilized to sustain this young patient, each adapted to manage the complexities of her prolonged treatment. These included four EUROSETS Alone Polymethylpentene (PMP), the Maquet PLS, Paragon PMP, four Terumo Fx15 oxygenators, and a Quadrox-i. Due to the extraordinary duration of ECMO support and the frequent complications encountered, including thrombosis and high transmembrane pressures, each oxygenator had varying durations of use. EUROSETS oxygenators were notably durable, with the third EUROSETS model (EUROSETS nr 3) lasting the longest at 88 days. In contrast, the Terumo Fx15 models had shorter durations, with some lasting only 1–2 days, primarily due to plasma leakage, which were recurrent issues with polypropylene-based oxygenators. Additionally, plasma leakage was particularly observed in the Terumo Fx15 oxygenators, necessitating more frequent replacements (Figure 2). The high LDH levels and elevated free Hb, particularly with the Quadrox-i and Terumo Fx15 models, indicate the hemolytic stress and ongoing coagulation challenges faced over the course of treatment. Continuous anticoagulation was maintained throughout the ECMO support, although three Terumo oxygenators were operated without anticoagulation to manage hemorrhage risks. The tables summarize the performance parameters and laboratory parameters observed across each oxygenator type during the treatment. Parameters such as Hb, PaO_2_ post-ox, PaCO_2_ post-ox, and FiO_2_ show the adaptation to each oxygenator’s functionality, with fluctuations reflecting the technical limitations and biological responses (Table 1). Table 2 summarizes the highest and lowest parameters from the scientific evidence for each oxygenator models presented as mean and deviation standard values. Figure 3 presents the graphical trend of the values in the different Oxygenator models.

**Table 2:** The highest and lowest parameters for each oxygenator models presented as mean and deviation standard values.

Oxygenator Model	Duration (Days)	Highest pO_2_ post-ox (Mean ± SD) (mmHg)	Lowest pO_2_ post-ox (Mean ± SD) (mmHg)	Highest pCO_2_ post-ox (Mean ± SD) (mmHg)	Lowest pCO_2_ post-ox (Mean ± SD) (mmHg)	Highest Air Flow (Mean ± SD) (L/min)	Lowest Air Flow (Mean ± SD) (L/min)	Highest Blood Flow Rate (Mean ± SD) (L/min)	Lowest Blood Flow Rate (Mean ± SD) (L/min)	Highest Pressure Drop (ΔP) (Mean ± SD) (mmHg)	Lowest Pressure Drop (ΔP) (Mean ± SD) (mmHg)
EUROSETS nr1	10	419 ± 34	215 ± 20	32 ± 5	52 ± 6	2.0 ± 0.3	2.5 ± 0.2	3.5 ± 0.5	3.2 ± 0.4	61 ± 5	67 ± 6
EUROSETS nr2	32	326 ± 19	207 ± 18	26.4 ± 4	40.2 ± 4	0.8 ± 0.1	4.0 ± 0.3	2.41 ± 0.3	1.7 ± 0.2	42 ± 4	75 ± 5
EUROSETS nr3	88	361 ± 27	216 ± 25	31 ± 4	39 ± 4	0.8 ± 0.1	4.0 ± 0.3	2.33 ± 0.3	1.9 ± 0.2	40 ± 4	75 ± 5
EUROSETS nr4	26	351 ± 19	224 ± 30	32 ± 5	38 ± 5	0.8 ± 0.1	5.0 ± 0.5	2.23 ± 0.2	0.5 ± 0.1	41 ± 5	170 ± 10
Maquet PLS	14	324 ± 20	60 ± 5	28 ± 3	42 ± 4	1.5 ± 0.2	8.0 ± 0.5	2.2 ± 0.3	2.0 ± 0.2	25 ± 3	75 ± 5
Paragon PMP	78	473 ± 45	230 ± 35	23 ± 3	37 ± 4	1.0 ± 0.2	2.0 ± 0.2	2.2 ± 0.3	2.0 ± 0.2	35 ± 4	57 ± 4
Terumo Fx15 nr1	5	287 ± 30	125 ± 15	30 ± 4	42 ± 4	2.0 ± 0.3	4.0 ± 0.3	2.31 ± 0.3	2.0 ± 0.3	31 ± 4	67 ± 5
Terumo Fx15 nr2	1	346 ± 40	138 ± 18	30.3 ± 3	34 ± 5	2.0 ± 0.3	3.0 ± 0.2	2.3 ± 0.2	2.2 ± 0.3	35 ± 5	40 ± 3
Terumo Fx15 nr3	3	321 ± 25	115 ± 14	32 ± 5	40 ± 4	2.0 ± 0.2	4.0 ± 0.3	2.4 ± 0.2	2.3 ± 0.2	32 ± 4	54 ± 5
Terumo Fx15 nr4	2	399 ± 30	98 ± 10	31 ± 4	38 ± 4	2.0 ± 0.3	4.0 ± 0.2	2.3 ± 0.2	2.2 ± 0.3	40 ± 5	41 ± 4
Quadrox-i	1	240 ± 35	121 ± 20	31 ± 3	40 ± 5	2.0 ± 0.3	4.0 ± 0.3	2.7 ± 0.3	2.3 ± 0.2	35 ± 4	50 ± 5

## Discussion

This case highlights the profound challenges of managing prolonged ECMO support in a resource-limited, conflict-affected environment. Prolonged ECMO support for 259 days is an exceptional scenario, particularly in a war-torn setting where logistical and material limitations compound the complexity of advanced medical care. The patient’s clinical course was complicated by underlying congenital heart disease, including severe aortic stenosis, and additional acute conditions such as myocarditis, respiratory distress, and bilateral pneumonia. The chronic nature of her cardiac dysfunction, combined with the war-impacted healthcare infrastructure, imposed substantial limitations on treatment options, most notably the lack of viable transplant alternatives [1, 2, 9]. The ECMO course involved several significant adaptations, including multiple transitions in ECMO configuration to match the patient’s shifting hemodynamic needs. Starting with VV-ECMO to support respiratory function, the configuration was converted to VA-ECMO as cardiac function deteriorated. This adaptability in ECMO configuration was critical for sustaining the patient over the extended treatment period, despite the associated risks and complications with each change [3]. Nevertheless, the prolonged duration of VA-ECMO support underscores the severity and persistence of her cardiac dysfunction, which was ultimately refractory to all attempts at weaning. The war-driven scarcity of medical supplies introduced unique challenges to ECMO management. Due to supply chain disruptions, the medical team was forced to use polypropylene-based oxygenators, including Quadrox-i and Terumo Fx15, which are generally unsuitable for long-term use due to the increased risk of clot formation [4, 5]. These oxygenators required frequent replacements due to mechanical failure, leading to increased procedural risks and heightened resource consumption. EUROSETS oxygenators demonstrated relatively better performance in terms of duration, with one oxygenator lasting up to 88 days. However, the frequent need for replacements underscores the limitations imposed by the lack of specialized, long-term ECMO-compatible oxygenators, and the extraordinary lengths the team had to go to adapt to the circumstances. Thrombosis and bleeding complications were recurrent throughout the ECMO course. High transmembrane pressures, indicative of clot formation, necessitated circuit and oxygenator replacements, while bleeding events such as pulmonary hemorrhage and epistaxis required intensive management [6, 7]. The patient developed hemorrhagic complications that escalated into a critical pulmonary hemorrhage on July 9, 2024, requiring selective bronchial occlusion to achieve hemostasis [8, 9]. These events were exacerbated by the absence of heparin during specific periods, a precaution taken to manage the bleeding [10, 11]. The frequent circuit changes, particularly in a setting where optimal ECMO equipment was not consistently available, further illustrate the complex interplay of thrombosis and bleeding management in prolonged ECMO support. Despite repeated attempts, weaning from ECMO was unsuccessful. Pulmonary function showed significant recovery, evidenced by normal PaCO_2_ and high PaO_2_ levels in subsequent tests, but cardiac function remained critically compromised [12]. The patient’s heart continued to exhibit severe dysfunction with a low EF, LVOT VTI, and other metrics indicating minimal contractile capacity. Although a heart-lung transplant was theoretically the best solution, the lack of a compatible donor and the limited transplant infrastructure in Ukraine, especially during wartime, rendered this option unfeasible. This limitation emphasizes the critical role that accessible transplantation services play in the management of end-stage organ failure and highlights the impact of conflict on healthcare resources and patient outcomes. In the challenging wartime context of Ukraine, ECMO therapy poses a unique set of logistical and medical hurdles, particularly concerning the availability of anticoagulants and advanced medical devices. Heparin remains the primary anticoagulant used in pediatric patients due to its relative availability, despite Bivalirudin being a preferable alternative for its reduced bleeding risk. Unfortunately, the latter is scarcely available and economically unfeasible given the current circumstances. Additionally, the use of advanced devices like the Pedimag (Levotronix) as a bridge to transplant was explored but remains inaccessible due to severe limitations in supply chains and financial constraints. These issues extend to the challenges of arranging patient transfers for transplantation to neighboring countries such as Latvia, Lithuania, or Poland, which are complicated by bureaucratic barriers, logistical difficulties, and the unpredictability of safe transport routes.

### Limitations

The data presented in this study also possess inherent limitations that must be acknowledged. The analysis is based on a single case, which limits the statistical power and broader applicability of the findings. Additionally, due to the exigencies of war, data collection was subject to interruptions and inconsistencies, potentially leading to gaps or biases in the recorded information. The reliance on a limited range of oxygenators, dictated by availability rather than optimal choice, may have influenced the outcomes and the subsequent analysis. Furthermore, the emergency nature of medical interventions during the conflict may have compromised the precision and reliability of clinical measurements, introducing an element of uncertainty in evaluating the efficacy of ECMO support. Recognizing these data limitations is crucial for interpreting the study’s conclusions and for planning future research in similar settings. The case illustrates the resilience and adaptability required from healthcare providers managing complex, high-risk treatments in conflict settings. The team’s capacity to maintain ECMO support for such an extended period, despite equipment shortages, frequent circuit and oxygenator replacements, and recurrent complications, demonstrates remarkable commitment to sustaining life [8, 9]. This case also underscores the pressing need for robust, war-resilient healthcare supply chains that can support advanced therapies, including ECMO, under adverse conditions. In conclusion, this case highlights both the technical challenges and the human resilience involved in managing prolonged ECMO in a conflict zone. Ultimately, the limitations imposed by the conflict environment significantly impacted the patient’s outcome, despite the best efforts of the medical team. Furthermore, the establishment of an international support system could significantly ameliorate the challenges faced in conflict zones. Such a system might include a collaborative network of healthcare providers, facilitated access to medical supplies via established logistics corridors, and shared guidelines for critical care practices. This support network could also incorporate training modules and real-time consultation services to assist local medical teams in managing complex cases with limited resources.

## Conclusions

This case highlights the critical role of oxygenator durability in sustaining prolonged ECMO support in conflict zones where resources are scarce. The 259-day ECMO course required frequent oxygenator replacements, driven largely by the limitations of polypropylene-based models like Terumo Fx15, which exhibited plasma leakage. The relative durability of the EUROSETS oxygenators, particularly one that lasted 88 days, proved essential in this setting, though even these models faced periodic issues with clotting and transmembrane pressure. In conflict-impacted environments, where the supply of specialized medical devices is unpredictable, durable oxygenators are vital for minimizing procedural risks and resource strain. This case underscores the need for resilient healthcare infrastructure and reliable access to long-term ECMO-compatible devices to support critically ill patients under challenging conditions.

## Data Availability

The data presented in this study are available on request from the Corresponding author.
